# Enhancing the Microparticle Deposition Stability and Homogeneity on Planer for Synthesis of Self-Assembly Monolayer

**DOI:** 10.3390/nano8030164

**Published:** 2018-03-14

**Authors:** An-Ci Shih, Chi-Jui Han, Tsung-Cheng Kuo, Yun-Chien Cheng

**Affiliations:** Department of Mechanical Engineering, National Chiao Tung University, Hsinchu 300, Taiwan; ancishih@gmail.com (A.-C.S.); dragon199357@gmail.com (C.-J.H.); lincecumepon@gmail.com (T.-C.K.)

**Keywords:** microparticle deposition, self-assembly, homogeneity, monomer synthesis, mask

## Abstract

The deposition stability and homogeneity of microparticles improved with mask, lengthened nozzle and flow rate adjustment. The microparticles can be used to encapsulate monomers, before the monomers in the microparticles can be deposited onto a substrate for nanoscale self-assembly. For the uniformity of the synthesized nanofilm, the homogeneity of the deposited microparticles becomes an important issue. Based on the ANSYS simulation results, the effects of secondary flow were minimized with a lengthened nozzle. The ANSYS simulation was also used to investigate the ring-vortex generation and why the ring vortex can be eliminated by adding a mask with an aperture between the nozzle and deposition substrate. The experimental results also showed that particle deposition with a lengthened nozzle was more stable, while adding the mask stabilized deposition and diminished the ring-vortex contamination. The effects of flow rate and pressure were also investigated. Hence, the deposition stability and homogeneity of microparticles was improved.

## 1. Introduction

Aerosol-based microparticle deposition technology is very commonly used for film fabrication process on a nanoscale [1,2,3,4], with several methods developed to deposit and pattern microparticles on substrates [5,6,7,8,9,10,11]. The advantages of aerosol-based microparticle deposition include cost effective equipment and the ability for microparticles to be made of various materials that are tailored to different applications. The applications of microparticle deposition include xerography printing [12], fuel-cell fabrication [13], electronic-element fabrication [14], film fabrication [15,16,17,18], peptide-array synthesis [19,20,21], etc. Among these applications, the particle-based peptide-array synthesis technique is a novel technique for microarray fabrication. It addresses solid amino-acid microparticles onto substrates and synthesizes high-density peptide arrays. Compared with other peptide array fabrication methods, the particle-based peptide array synthesis has higher spot density than SPOT synthesis [22] and is faster than photolithography synthesis [23,24].

The aerosol for particle-based peptide array synthesis was generated using compressed air, with the microparticles triboelectrically charged in turbulence. The charged microparticles will be attracted to the electrical field generated by microelectrodes, before being deposited on the microelectrode array in the desired pattern [19,25]. However, the same turbulence that generates aerosol and triboelectrically charges the microparticles also result in inhomogeneity in the microparticle deposition. Inhomogeneous microparticle deposition leads to unequal amounts of synthesized peptide, which decreases the reliability of array analysis. Hence, stabilizing the turbulence to create homogeneity in the microparticle deposition becomes an important issue. Although several studies have discussed how to pattern the microparticle deposition [5,7,8,9,10,26,27], stabilizing the aerosol turbulence for homogeneous microparticle deposition has not yet been fully investigated.

In this study, the turbulence was stabilized with lengthened aerosol nozzles and masks. A two-dimensional flow field simulation was used to investigate the ring vortex generated and to explain why the mask can eliminate ring vortex. Furthermore, we also enhanced the microparticle deposition stability and homogeneity by optimizing flow rates and pressures associated with lengthened nozzles and masks. The results in this study will help to improve the deposition homogeneity of particle-encapsulated monomers and hence, enhance the homogeneity of self-assembly synthesized peptide nanolayers.

## 2. Experimental and Simulation Setup 

### 2.1. Aerosol Generation and Deposition System

**Figure 1:**
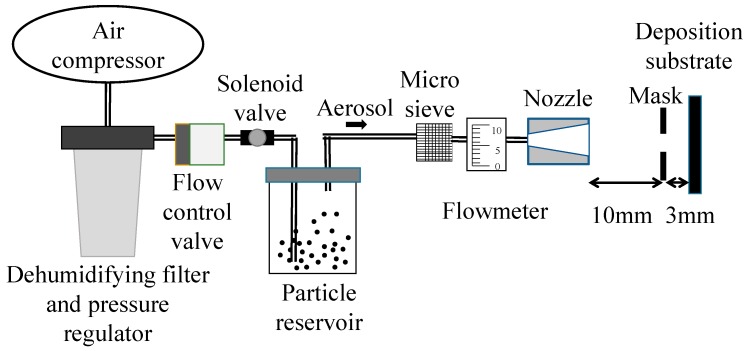
Aerosol generation and deposition system.

In this study, the aerosol generation and deposition system was developed by referencing previous studies [25,28], which is shown in Figure 1. The system included an air compressor (JW-2525N, JUN WEI, Taichung, Taiwan), dehumidifying filter and pressure regulator (MATFR401, Mindman, Taipei, Taiwan), flow control valve (GRLSA-1/8-QS-6, Festo, Esslingen am Neckar, Germany), solenoid valve (MHJ10-S-0,35-QS-4-MF, Festo, Esslingen am Neckar, Germany), microparticle reservoir (Falcon™ 50 mL conical centrifuge tube, Corning, New York, NY, USA), micro sieve, flowmeter and our custom-made nozzle. The nozzles were divergent pipes to allow for stabilization of the flow [29,30,31]. Polytetrafluoroethylene (PTFE) tube was chosen to connect the aerosol system for the triboelectrical charge. Toner microparticles (Black, Xerox, Norwalk, CT, USA) were used for microparticle deposition experiments. For the particle size, 50% of the particles were smaller than 6.016 μm and 90% of particles were smaller than 8.309 μm [32]. Xeros Black particles were used in the study because the particle size is similar to the expensive bioparticles [33]. The result of this study will be applied to create homogeneity in bioparticle [11] deposition in the future. The deposited bioparticles can be addressed in different methods and used for monomer synthesis, such as electrical fields from microelectrodes [19,25] or laser [34,35,36,37], and can be used for several applications, such as antibody analysis [38] or surface functionalization [39]. The microparticles were dehumidified in the desiccator with a molecular sieve overnight before the experiment. 

To generate the microparticle aerosol, the high pressure air was generated by the air compressor, which passed through the dehumidifying filter and pressure regulator. The air flow was controlled using a flow control valve and solenoid valve. Following this, the regulated air flow entered the microparticle reservoir and generated a microparticle aerosol with turbulence. After the agglomerated microparticles were broken up by passing through the micro-sieve, the microparticles were deposited on the substrate. In this study, the effect of including the mask between the nozzle and substrate was discussed. 

### 2.2. Measurement and Analysis of Microparticle Deposition

To analyze the stability and homogeneity of the microparticle deposition, the area, symmetric value and thickness of microparticle deposition were measured. The standard deviations σ of these indexes were considered to be indexes of deposition stability in this study. In addition, if the deposition is stable and symmetrical, the deposition homogeneity can be easily improved by moving the deposition location. The area of microparticle deposition was calculated with Photoshop (Adobe Systems Incorporated, San Jose, CA, USA). The pixel number of black (microparticle) and blank (chip) area were calculated and converted to mm^2^ according to Equation (1):

(1) $$\mathrm{Particle}\;\mathrm{depopsition}\;\mathrm{area}=\frac{n_{\mathrm{Part}}}{n_{\mathrm{Part}}+\;n_{\mathrm{Chip}}}\times 400\;{\mathrm{mm}}^{2},$$

where *n*_Part_ represented the pixel number of microparticle area and *n*_Chip_ represented the pixel number of chip area.

**Figure 2:**
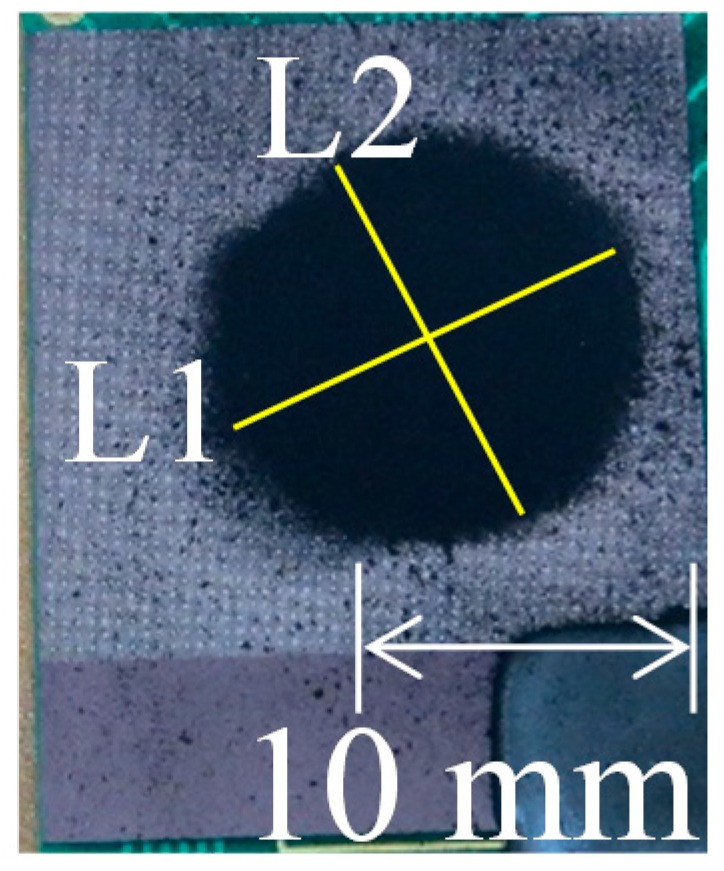
Symmetric value measurement of the deposited particle pattern where *L*1: longest axis and *L*2: shortest axis.

The symmetric value was evaluated by dividing the longest axis (*L*1) of the microparticle deposition (Figure 2) by its shortest axis (*L*2), which is shown in Equation (2).

(2) Symmetric value=L1/L2.

### 2.3. Simulation Method

The ANSYS FLUENT software (ANSYS, Canonsburg, PA, USA) was used to simulate the flow field in nozzles with different lengths and the flow field of deposition with or without mask. The aerosol flow field was considered as a single phase flow to simplify the simulation. The *k*-*ε* model was used to simulate the flow field. Equation (3) shows the continuity equation, which describes the mass that enters minus the mass that leaves the system. This is equal to the accumulation of mass in the system where *ρ* is fluid density; *U* is the flow velocity vector field; *t* is time; and *x* is the length. Equation (4) shows the momentum equation, which describes the relationship between acceleration of the gas and the force where *S*_M_ is the sum of body forces; $p^{\prime }$ is a modified pressure; and $\mu_{\mathrm{eff}}$ is the effective viscosity accounting for turbulence. Equation (5) shows the Boussinesq buoyancy model in the *k*-*ε* model, which is used to predict the buoyancy effects on production and destruction of turbulence where *P*_kb_ is the buoyancy production term; $\mu_{t}$ is viscosity; *ρ* is fluid density; $\sigma_{p}$ and β are constants; *g_i_* is acceleration in *x_i_* direction; and *T* is temperature.

(3) $$\frac{\partial \rho }{\partial t}+\frac{\partial \left(\rho U_{j}\right)}{\partial x_{j}}=0,$$

(4) $$\frac{\partial \rho U_{i}}{\partial t}+\frac{\partial \left(\rho U_{i}U_{j}\right)}{\partial x_{j}}=-\frac{\partial p^{\prime }}{\partial x_{i}}+\frac{\partial }{\partial x_{j}}\left[\mu_{\mathrm{eff}}\left(\frac{\partial U_{i}}{\partial x_{i}}+\frac{\partial U_{j}}{\partial x_{j}}\right)\right]+S_{M},$$

(5) $$P_{\mathrm{kb}}=-\frac{\mu_{t}}{\rho \sigma_{\rho }}\rho \beta g_{i}\frac{\partial T}{\partial x_{i}}.$$

## 3. Results and Discussion

### 3.1. Nozzle Length and Flowrate Effects on the Stability of Deposited Microparticle Layer

**Figure 3:**
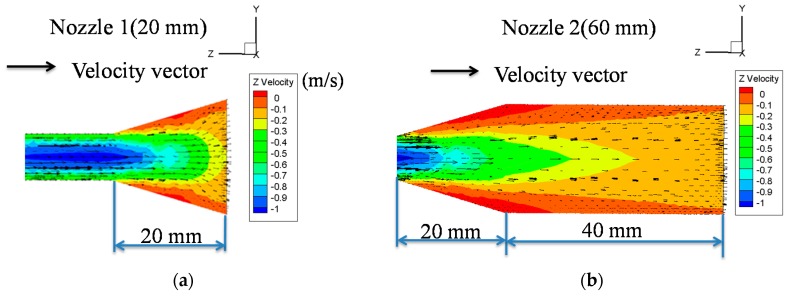
Fluid field simulation of different nozzle length, which was simulated by Fluent using: (**a**) 20 mm nozzle; and (**b**) 60 mm nozzle.

In the simulation, the nozzles were divergent pipes according to the experimental setup. The nozzle length was set to 20-mm and 60-mm. The speed of air flow at nozzle inlet was 1 m/s and the pressure at the nozzle outlet was 0. The simulation results are shown in Figure 3. The flow direction was from left to right, so the speed values were shown as negative values. 

Figure 3a shows the simulation results of the 20-mm nozzle. When the air flow entered the divergent pipe, the flow slowed down. The boundary layer was formed along the inner wall of the divergent pipe due to flow viscosity. Flow along the inner wall influenced the flow closer to the nozzle center and formed a secondary flow. About halfway through the divergent pipe, the flow speed near the inner wall was close to zero, which might have occurred due to the formation of small vortexes. The simulation results also showed that the flow speed at the nozzle outlet was between −0.3 and −0.1 m/s.

Figure 3b shows the simulation results of the 60-mm nozzle. When the air flow entered the divergent pipe, the flow also slowed down. Since the nozzle was lengthened, the influence of boundary layer on inner flow increased. Therefore, the flow speed at nozzle outlet was between −0.2 and −0.1 m/s. The simulation shows that the flow-velocity distribution of 60-mm nozzle was more uniform than that of 20-mm nozzle. The flow direction of 60-mm nozzle was also more consistent than that of 20-mm nozzle. Hence, using 60-mm nozzle can result in more stable outlet flow compared to using 20-mm nozzle.

For the nozzle length experiment, the parameters used in the experiment were the same as those used in simulation. The air flow pressure, flow rate and distance between nozzle and substrate were 0.1 MPa, 5 L/min and 10 mm, respectively. Based on the flow rate and cross section of nozzle, the flow speed was calculated as 1 m/s. 

**Figure 4:**
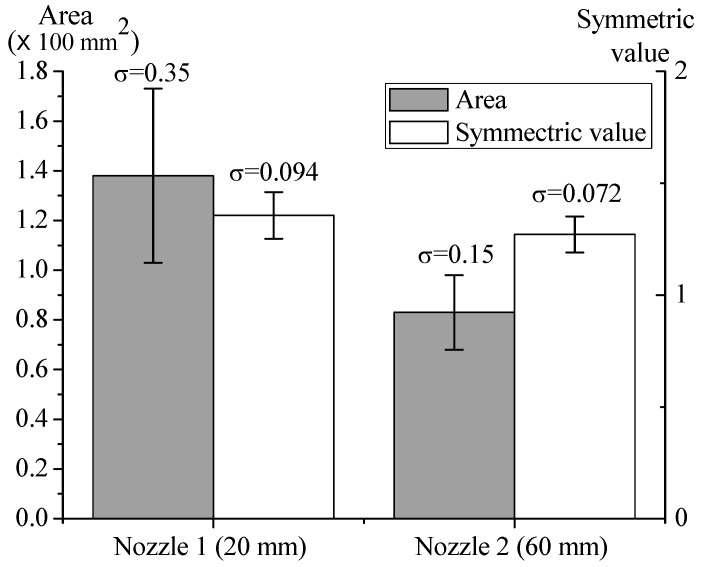
Effects of nozzle length on particle deposition. Data are shown with mean ± SD (*n* = 3).

The experimental results are shown in Figure 4. For the 20-mm nozzle, the average microparticle deposition area, standard deviation (σ) of deposition area and symmetric value were 137.683, 35.025 and 1.237 mm^2^, respectively. For the 60-mm nozzle, the microparticle deposition area, σ of deposition area and symmetric value were 83.18, 15 and 1.152 mm^2^, respectively. The standard deviation of the deposition area with the 60-mm nozzle was smaller than that with 20-mm nozzle, which indicated greater deposition stability of 60-mm nozzle over 20-mm nozzle. Furthermore, the symmetric value of the deposition area with 60-mm nozzle was smaller than that with 20-mm nozzle, which also means that the 60-mm-nozzle deposition was more symmetric and stable. This result supports the simulation result that the 60-mm nozzle can stabilize the air flow and thus, the deposition.

**Figure 5:**
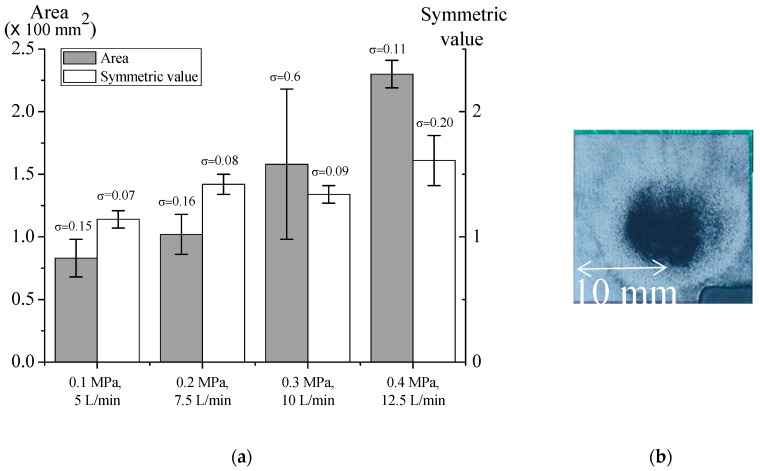
(**a**) Effects of pressure and flow rate on particle deposition with 60-mm nozzle. Data are shown with mean ± SD (*n* = 3); and (**b**) Ring vortex.

Since the 60-mm nozzle resulted in more stable microparticle deposition, we investigated the pressure and flow rate effects on microparticle deposition with a 60-mm nozzle. The results are shown in Figure 5a. Both deposition area and symmetric value increased with the flow rate. The increase of flow rate resulted in turbulence, making the deposition unstable. Furthermore, the ring vortex (Figure 5b) appeared in every deposition. The pattern of ring vortex in deposition center was round, but there was a ring-shaped pattern outside. This is because the microparticles were blown away from the center. The ring vortex will contaminate the deposition area and decrease the homogeneity of the deposition.

### 3.2. Mask for Removing the Ring Vortex and Enhancing Deposition Stability

To solve the ring vortex problem occurring during deposition, a mask with fixed aperture was placed between nozzle and substrate, with the effects discussed with simulation and experiments. 

**Figure 6:**
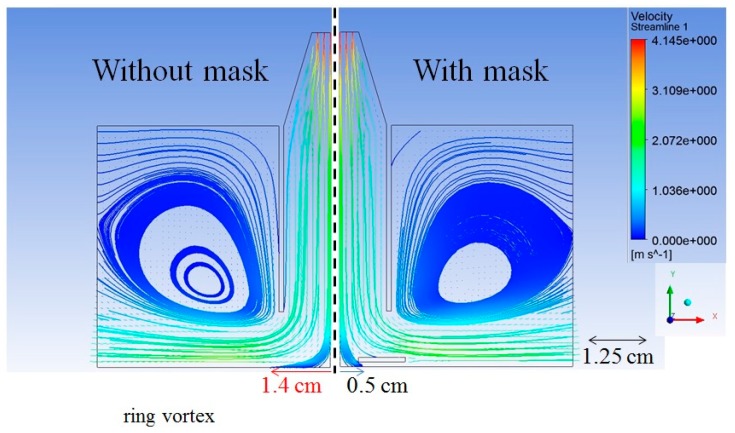
Simulation of flow field on the chip surface with and without mask.

The flow fields with and without mask using the 60-mm nozzle were simulated. The flow speed at the nozzle inlet was 4.145 m/s, which was calculated from the 12.5 L/min volume flow rate. The simulation results were shown in Figure 6. As the flow fields in our study are symmetric, we joined two simulations into one diagram for more intuitive side-by-side comparison. The left panel displays the simulation without mask, while the right displays the simulation with mask. The streamlines of flow field could be considered as the moving tracks of microparticles.

Without using the mask, when the air flow reached the substrate, the inner flow stopped at the surface and the outer flow formed the ring vortex. The distribution area of flow field at the substrate was smaller with the mask than without due to partial blockage of the flow by the mask. The part of flow that generated the ring vortex was also blocked. Therefore, adding the mask resulted in smaller but more stable microparticle deposition. For the speed of flow field, the flow speed was faster in the periphery (2−3.1 m/s) than that close to the center. Therefore, the microparticles could be easily dispersed in the peripheral outer area. With the mask on the substrate, the dispersion of microparticles in the outer area was blocked and most of the deposited microparticles resulted from the stable flow in the inner area.

**Figure 7:**
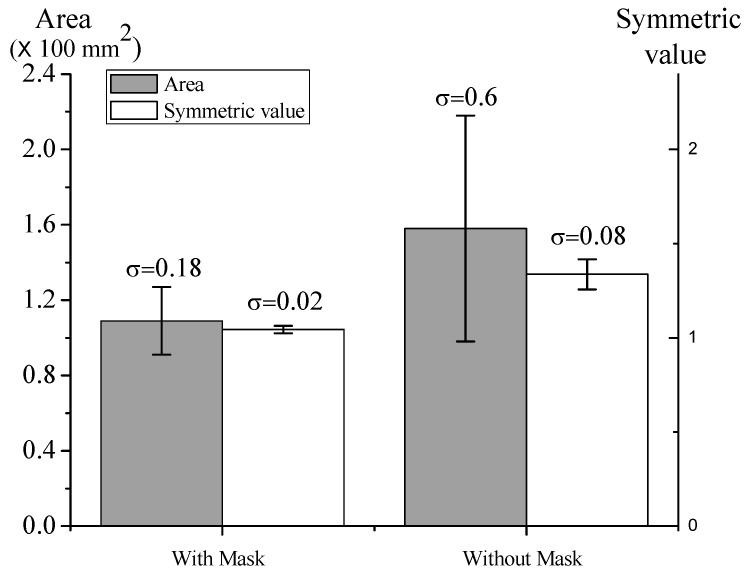
Particle deposition stability with and without mask. The pressure was 0.3 MPa; flow rate was 10 L/min; and distance was 10 mm. Data are shown with mean ± SD (*n* = 3).

The effect of masking was also examined with actual experiments. A mask with a round aperture (radius of 4 mm) was placed between the nozzle and deposition substrate, which is shown in Figure 1. The air pressure was 0.3 MPa and the flow volume was 10 L/min. The deposition area, symmetric value and standard deviation (σ) are shown in Figure 7. With the mask, the average deposition area, σ of deposition and symmetric value were respectively 109.33, 18 and 1.044 mm^2^. Without the mask, the respective values were 157.67, 60 and 1.337 mm^2^. The smaller σ of deposition area indicates that the deposition with the mask was more stable. The symmetric value with mask was closer to 1, which also means that the deposition was more stable than the deposition without the mask. The difference between area with and area without the mask was not significant.

### 3.3. Effects of Mask Size, Pressure, and Flow Rate on Microparticle Deposition Stability with Mask

Since the mask can stabilize the microparticle deposition, the effects of mask size, pressure and flow rate on microparticle deposition with the mask were investigated to further enhance the deposition stability.

#### 3.3.1. Effects of Mask Size

**Figure 8:**
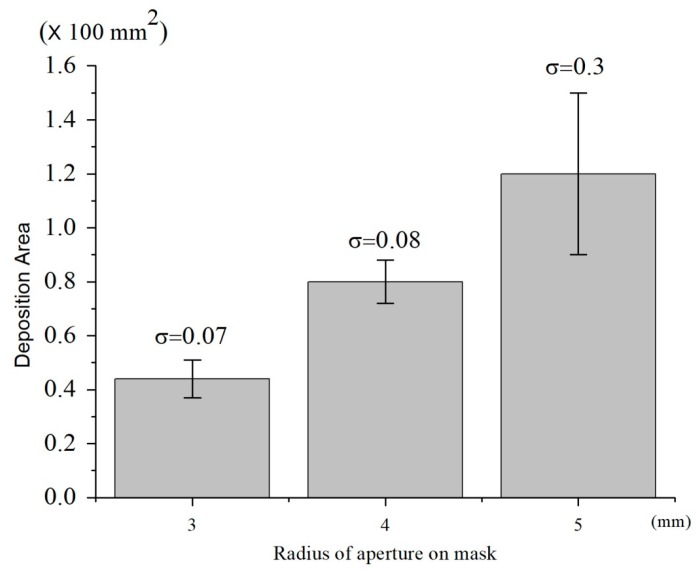
Effects of aperture size on particle deposition stability. Data are shown with mean ± SD (*n* = 3).

The experimental results of microparticle deposition with different aperture sizes were shown in Figure 8. For the aperture radii of 3−5 mm, the average deposition area was 44.529, 80.099 and 120.105 mm^2^, while the σ of deposition was 7.131, 8.116 and 30.045. The air flow through the mask still generated minor turbulence between the mask and substrate. The amount of air that passed through increased with mask aperture, with a corresponding increase in turbulence. The small mask aperture allowed less air to pass, with more stable and smaller deposition. 

#### 3.3.2. Effects of Pressure and Flow Rate

**Figure 9:**
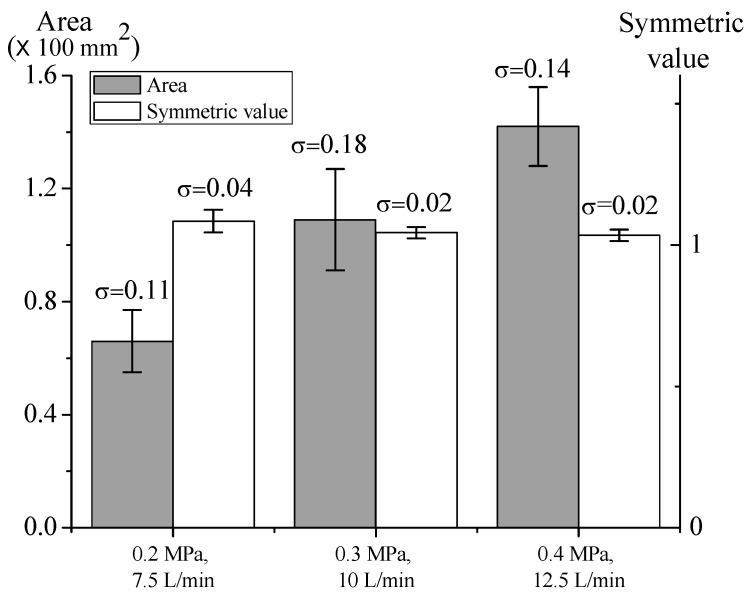
Effects of different pressures and flow rates on particle deposition with mask. Data are shown with mean ± SD (*n* = 3).

The aperture with a 3 mm radius resulted in most stable microparticle deposition. Hence, we investigated pressure and flow rate effects on deposition with a 3 mm aperture (Figure 9) and also compared the results against deposition without masks (Figure 5). The symmetric value of deposition with mask was closer to 1 than that without mask, so the mask did stabilize the microparticle deposition. On the other hand, the higher pressure and flow rate resulted in larger deposition area, since more microparticles were pumped out by higher pressure. 

### 3.4. Particle Deposition in Initial Condition and after Optimization

**Figure 10:**
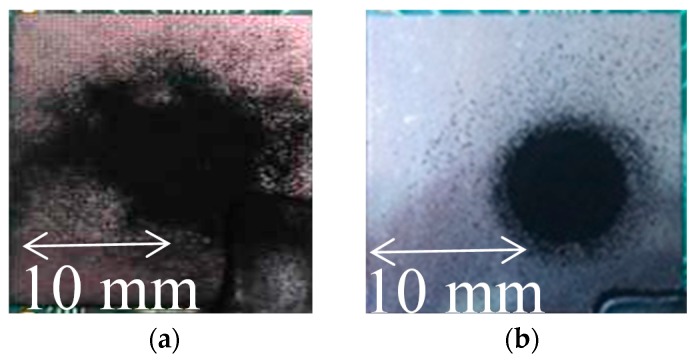
Particle deposition before and after optimization: (**a**) without mask at a flow rate of 12.5 L/min and (**b**) using mask with 6 mm aperture at a flow rate of 7.5 L/min.

Figure 10 shows the particle deposition under the initial conditions and after optimization. The deposition with a 6 mm aperture mask and low flow rate (Figure 10b) is more symmetric and stable than the deposition without the aperture and high flow rate. 

## 4. Conclusions

The experiment and simulation results showed that a longer nozzle can reduce the effect of secondary flow and can make the microparticle deposition stable and thus, homogeneous. Increasing the pressure and flow rate generated turbulence and ring vortex, resulting in instability in the deposition. Using the mask can prevent the ring vortex from contaminating the deposition and stabilize the deposition. The mask with a 6 mm aperture had the most stable deposition, since there was more turbulence generated than when other masks were used. With a 6 mm aperture, decreasing the pressure or decreasing the flow rate increased the deposition stability. The experimental conditions that had the most stable deposition used a mask with a 6 mm aperture, 5 L/min flow rate and 0.15 MPa pressure. These results can facilitate the applications that need stable and homogeneous microparticle deposition, which will thus ensure the uniformity of microparticle-based nanolayer synthesis.
